# Autophagy: A challengeable paradox in cancer treatment

**DOI:** 10.1002/cam4.5577

**Published:** 2023-02-09

**Authors:** Farnaz Ahmadi‐Dehlaghi, Parisa Mohammadi, Elahe Valipour, Pouya Pournaghi, Sarah Kiani, Kamran Mansouri

**Affiliations:** ^1^ Medical Biology Research Center, Health Technology Institute Kermanshah University of Medical Sciences Kermanshah Iran; ^2^ Department of Biology Payame Noor University Tehran Iran; ^3^ Department of Tissue Engineering and Applied Cell Sciences, School of Advanced Technologies in Medicine Shahid Beheshti University of Medical Science Tehran Iran; ^4^ Department of Medical Genetics, School of Medicine Tehran University of Medical Sciences Tehran Iran; ^5^ Medical Biology Research Center Kermanshah University of Medical Sciences Kermanshah Iran

**Keywords:** angiogenesis, autophagy, cancer biology, cancer management, drug design, signal transduction

## Abstract

**Objective:**

Autophagy is an intracellular degradation pathway conserved in all eukaryotes from yeast to humans. This process plays a quality‐control role by destroying harmful cellular components under normal conditions, maintaining cell survival, and establishing cellular adaptation under stressful conditions. Hence, there are various studies indicating dysfunctional autophagy as a factor involved in the development and progression of various human diseases, including cancer. In addition, the importance of autophagy in the development of cancer has been highlighted by paradoxical roles, as a cytoprotective and cytotoxic mechanism. Despite extensive research in the field of cancer, there are many questions and challenges about the roles and effects suggested for autophagy in cancer treatment. The aim of this study was to provide an overview of the paradoxical roles of autophagy in different tumors and related cancer treatment options.

**Methods:**

In this study, to find articles, a search was made in PubMed and Google scholar databases with the keywords Autophagy, Autophagy in Cancer Management, and Drug Design.

**Results:**

According to the investigation, some studies suggest that several advanced cancers are dependent on autophagy for cell survival, so when cancer cells are exposed to therapy, autophagy is induced and suppresses the anti‐cancer effects of therapeutic agents and also results in cell resistance. However, enhanced autophagy from using anti‐cancer drugs causes autophagy‐mediated cell death in several cancers. Because autophagy also plays roles in both tumor suppression and promotion further research is needed to determine the precise mechanism of this process in cancer treatment.

**Conclusion:**

We concluded in this article, autophagy manipulation may either promote or hinder the growth and development of cancer according to the origin of the cancer cells, the type of cancer, and the behavior of the cancer cells exposed to treatment. Thus, before starting treatment it is necessary to determine the basal levels of autophagy in various cancers.

## 1 INTRODUCTION

Autophagy is an evolutionarily conserved degradation process that helps maintain cell homeostasis and survival under normal and stressful conditions. In this intracellular catabolic pathway, damaged cytoplasmic components transported into lysosomes are degraded by lysosomal acid hydrolase enzymes; resulting ingredients such as amino acids, fatty acids, sugars, and nucleotides are released into the cytosol, and then used for cellular metabolism.^1^, ^2^

Three main types of autophagy have been described: micro‐autophagy, Chaperone‐mediated autophagy (CMA), and macro‐autophagy. The micro‐autophagy pathway involves trapping and eliminating damaged and redundant cytosolic components directly by the lysosomal membrane.^3^, ^4^ This pathway also plays a role in maintaining organelle size and membrane composition.^4^ In the CMA, excess and damaged proteins with a specific KFERQ motif are transported close to the lysosomal membrane by heat shock protein 70 (HSC‐70) and its co‐chaperones, which are then transported into the lysosomes through interacting with a lysosomal membrane protein called lysosome‐associated membrane protein 2A (LAMP2A).^4^, ^5^ The CMA seems to be more involved in starvation responses by playing key roles in the removal of oxidized and misfolded proteins.^4^ In the macro‐autophagy pathway (referred to as autophagy hereinafter), damaged cellular components are engulfed by double‐membrane vesicles (so‐called autophagosome). These vesicles fuse with and deliver their cargo to lysosomes, where they are broken down by the activities of various hydrolases. The degraded substances are released into the cytosol and used for cellular metabolism.^6^ Non‐selective autophagy and cargo‐specific autophagy (also called: mitophagy, ribophagy, pexophagy, and xenophagy) are two types of macro‐autophagy.^7^

In the absence of stressful conditions, the cell utilizes baseline levels of autophagy to support biological functions, maintain metabolic homeostasis, quality‐control of cell contents, and eliminate long‐lived or misfolded proteins and damaged organelles.^8^, ^9^ Nevertheless, autophagy is induced following fluctuations in oxygen and nutrients (stressful conditions) to support survival and normal cellular metabolism.^9^, ^10^

There are several studies showing the involvement of autophagy defects in different human diseases, including cancer.^4^, ^11^ In addition, autophagy appears to be able to play paradoxical roles in cancer progression. Thus, autophagy has been suggested to counteract the onset of cancer in the early stages of tumor formation. In an established tumor, however, this process supports the survival of tumor cells by helping them adapt to stressful conditions such as lack of oxygen and nutrients.^4^, ^11^ Also, Santana‐Codina et al. report suggested in a study that autophagy may be necessary to maintain tumor survival depending on the tumor type and source tissue.^12^

Although autophagy and its manipulation have been considered as a practical therapeutic target in the field of cancer treatment^11^, ^13^ because most anti‐cancer therapies induce autophagy in tumor cells, it is not clear whether autophagy in cancer cells favors survival or death.^14^ In response to anti‐cancer therapies, there are various reports that autophagy acts like a double‐edged sword, in favor of cancer cells in some tumors and against tumor cell survival in others.^13^

Therefore, considering the paradoxical roles of autophagy in response to cancer therapies, two important questions arise; what are the initial and post‐treatment levels of autophagy in different tumors? What is the appropriate treatment option; induction or inhibition of autophagy? Here, we first overview the mechanisms and genes involved in autophagy and then address the paradoxical roles of autophagy in the development and progression of cancer, where it is highlighted as an anti‐cancer therapeutic target.

## 2 MOLECULAR MECHANISM OF AUTOPHAGY

To date, more than 30 autophagy‐related genes and proteins (ATG for autophagy‐related genes and Atg for their proteins) have been identified in yeast.^15^, ^16^ These genes are evolutionarily highly conserved, as they are present in higher eukaryotes and orthologs have been identified for them in humans.^16^ The process of autophagy consists of several steps:

1. The initial step involves inducing phagophore membrane formation.
2. Nucleation; engulfing cytoplasmic components by phagophore membrane that forms a cup‐shaped structure.
3. Elongation/expansion; phagophore membrane extends to be able to surround cytoplasmic components. The resulting structure is called an autophagosome, which is a structure with a bilayer membrane.
4. Fusion and degradation; the autophagosomes fuse with lysosomes to form autolysosomes (often called autophagolysosomes) and then, their cargos are destroyed by lysosomal hydrolase. The resulting components are released into the cytosol to be used as cellular energy sources.^7^, ^15^, ^17^

The autophagic flux can be triggered by intrinsic and extrinsic stresses such as starvation, hypoxia, growth factors deficiency, ATP/AMP ratio, intracellular Reactive oxygen species (ROS) rates, pathogens, or chemical drugs.^18^ In yeast, activation of intracellular signaling pathways in response to stressful conditions recruits Autophagy‐related genes ATGs to a subcellular location called the phagophore assembly site.^19^, ^20^ In mammalians, phagophore is primarily formed at a subdomain of Endoplasmic reticulum (ER) membrane called omegasome, which is a lipid bilayer membrane enriched for Phosphatidylinositol 3‐phosphate (PI3P).^21^

In mammalians, the Unc‐51‐like kinase (ULK) complex, which consists of ULK1/2 (homolog of yeast ATG1), FAK family‐interacting protein of 200 kDa (FIP200), Atg13, and Atg101, is responsible for the initiation of phagophore formation.^15^, ^22^ The initial step is regulated by mammalian target of rapamycin (mTOR) and AMP‐activated Protein Kinase (AMPK) pathways. Under normal conditions and in the presence of sufficient amino acids and growth factors, Phosphatidylinositol 3‐kinase complex 1 (PI3KC1) activates the mTORC1 subunit, leading to the activity of mTOR complex and thereby prevention of the autophagy initiation through phosphorylation of two subunits of ULK complex, ULK1 and Atg13. In starvation conditions, mTORC1 is separated from the initiation complex, resulting in the ULK complex activation.^15^, ^22^ In fact, ULK1 phosphorylates and activates Atg13, Atg101, and FIP200. After activation, ULK complex localizes into the expanding phagophore membrane.^22^ The initiation complex is also induced by activation of AMPK which suppresses mTORC1. AMPK, in turn, is activated by glucose restriction and decreased ATP/AMP ratio.^8^, ^23^, ^24^

In the nucleation step, ULK1 protein phosphorylates and activates a class ІІІ phosphatidylinositol 3‐kinase (PI3KC3) complex. This complex consists of Bcl‐2‐interacting myosin‐like coiled‐coil (Beclin1), Vacuolar protein sorting 34 (Vps34), Vps15, and Atg14L.^11^ Beclin1 protein is a substrate for factors involved in the nucleation step.^5^ PI3KC3 complex also generates local PI3P in phagophores which is necessary for phagophore nucleation. PI3P promotes the recruitment of WD‐repeat domain phosphoinositide‐interacting 2 (WIPI2), which in turn recruits Atg12–Atg5–Atg16L complex for phagophore elongation.^21^ Proteins involved in the initiation and nucleation participate in the formation of phagophore membrane,^11^ which can be formed and expanded from plasma membrane, ER, mitochondria,^11^, ^25^ or Golgi apparatus.^24^, ^25^

**FIGURE 1:**
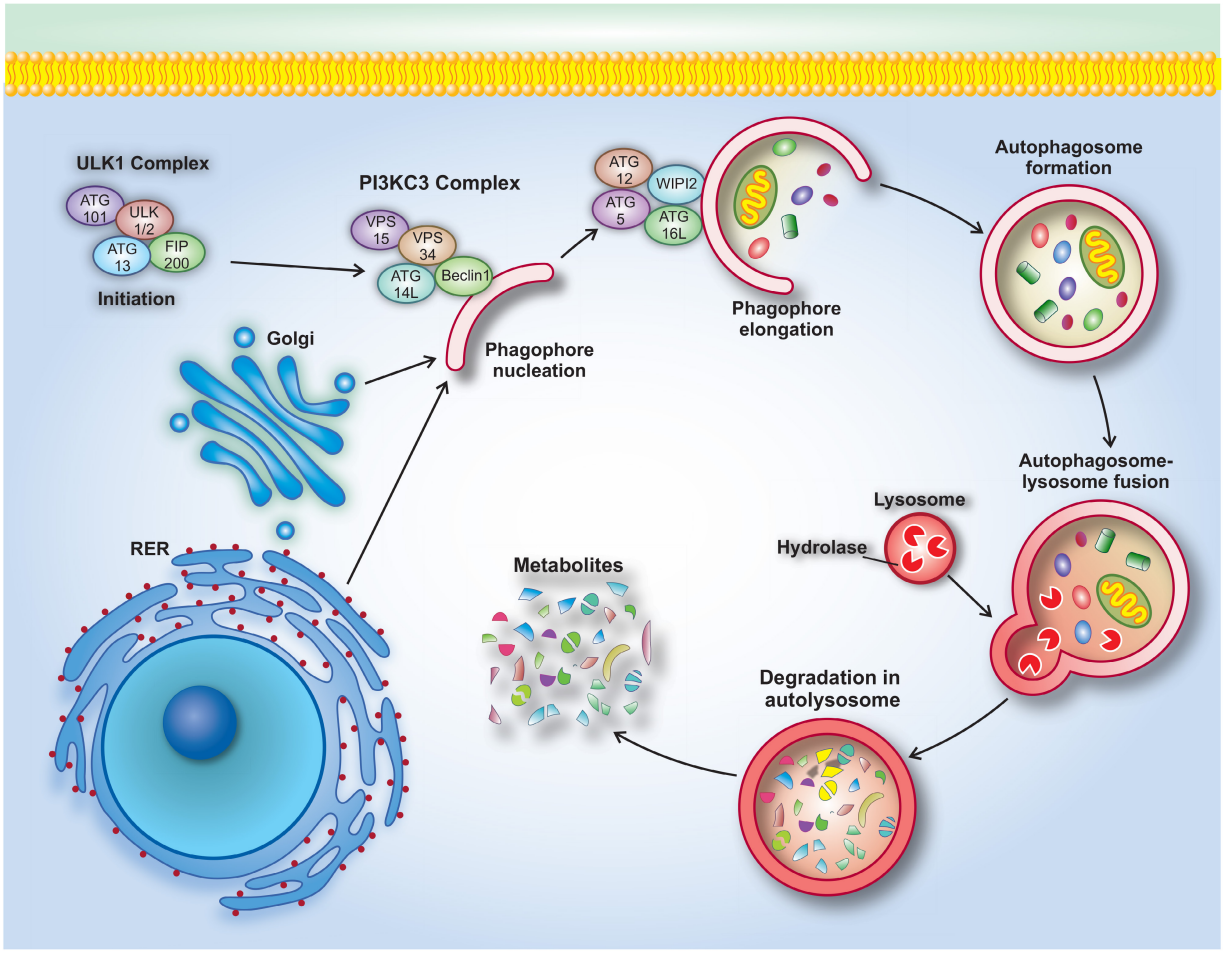
Autophagy process consists of several *sequential* steps. (1) Initiation. (2) Phagophore nucleation. (3) Phagophore elongation. (4) Fusion of autophagosome with lysosome and degradation of cargo in autolysosome.

In the elongation step, two ubiquitin‐like conjugation systems involved in the expansion and closure of phagophore membrane are described^4^, ^26^: In the first system, Atg12 is activated and conjugated to Atg5. Atg7 (E1 ligase) is involved in Atg12 activation, and Atg10 (E2 ligase) helps Atg12‐Atg5 conjugation.^22^, ^27^ Atg12‐Atg5 is then associated with Atg16L1 and hence autophagy elongation complex is formed, which provides a location for Lipidates microtubule‐associated protein 1 light chain 3 (Lc3) lipidation.^4^, ^22^ Binding of WIPI2 to Atg16L allows the Atg12‐Atg5‐Atg16L complex to be located on the autophagosome membrane.^27^ In the second system, Lc3 (also known as ATG8) through cleavage by Atg4B is converted to Lc3‐І, which in turn is conjugated with Phosphatidylethanolamine (PE) in the cytoplasm to produce Lc3‐ІІ. Then, Lc3‐ІІ is deposited in the autophagosome membrane which causes cargo recognition.^4^, ^5^, ^22^ Lc3‐ІІ also serves as an autophagosome marker and is essential for autophagosome maturation.^5^, ^26^, ^27^ After maturation and closure, the autophagosome fuses with lysosome, which ultimately leads to the degradation of autophagosome cargo. Several proteins are involved in the fusion step, such as Ras‐associated binding proteins (Rabs), Soluble N‐ethylmaleimide‐sensitive factor attachment proteins (SNAREs), and tethering complexes^27^ (Figure 1).

Dysfunction of autophagy has been described in various diseases including metabolic disorders, pulmonary disease, vascular diseases,^25^ abnormalities,^28^ neurodegenerative diseases, infections, cancers, and various myopathies.^27^, ^29^, ^30^ There is different evidence that autophagy exerts paradoxical roles in cancer, so that the role of autophagy in tumorogenesis is one of the most controversial issues in the field of cancer.^22^

## 3 PARADOXICAL ROLE OF AUTOPHAGY IN CANCER

The autophagy pathway is essentially considered a protective pathway of the cell against metabolic stress, DNA damage, and tumorogenesis by removing misfolded proteins, damaged organelles, and ROS. Various studies have suggested that autophagy plays a cytoprotective role in the early stages of tumor formation.^22^, ^27^ However, after tumor establishment and transformation of healthy cells into tumors and progression to advanced stages of cancer, increased autophagy has been reported that works in favor of resistance of tumor cells to stressful conditions such as hypoxia, nutrient deficiency, metabolic stress, chemotherapy, resulting in tumor survival,^17^, ^19^ proliferation and metastasis.^26^, ^31^

It is noteworthy that the dependence of tumor cells on autophagy varies in different tumors. While autophagy increases in several tumors such as pancreatic cancer, even in nutrient‐rich conditions,^32^ there are inconsistencies in a variety of cancers in which the level of autophagy in cancer cells is compared to that in non‐tumor cells.^33^ In addition, several studies have suggested that the effect of autophagy on the destiny of tumor cells depends on the type of tumor, genetic context, tumor stage,^19^, ^27^ and tumor microenvironment.^19^

Considering the roles of autophagy in maintaining genomic stability and preventing tumor formation in the early stages of cancer along with supporting the survival of tumor cells against various stresses in advanced stages of cancer,^7^ and also because of its paradoxical effects in treatment cancer (discussed in other sections), autophagy is referred to as the “double‐edged sword” in various studies.^34^

## 4 ROLE OF AUTOPHAGY IN TUMOR SUPPRESSION

There are several studies that have suggested a tumor suppressor role for autophagy. Tumor suppressor genes such as Phosphatase and tensin homolog (PTEN), P53, Tuberous sclerosis protein 1 and 2 (TSC1/2), and Death‐associated protein kinase (DAPk) induce autophagy and some autophagy suppressor genes such as Rat sarcoma virus (RAS) and AKT display oncogenic activities.^35^ Also, down‐regulation and loss of function of some Atgs have been reported in several cancers, suggesting possible tumor inhibitory functions for autophagy.^36^, ^37^ Takamura et al. indicated that mice with autophagic defects induced by systemic mosaic elimination of *ATG5* and specific deletion of *ATG7* showed benign adenomas in tissues such as liver and pancreas.^38^ In addition, Karantza‐Wadsworth et al. showed increased tumorogenesis of mammary cell lines with impaired autophagy through Beclin1 allelic loss.^39^ Increased susceptibility of Beclin1^+/−^ mice to Hepatocellular carcinoma (HCC), lung carcinoma and lymphoma has also been reported.^40^ Liang et al. demonsrated that normal breast epithelia frequently express endogenous Beclin1 protein at high levels, wherease human breast epithelial carcinoma cell lines and tissue frequently express endogenous Beclin1 protein al low levels.^41^ Moreover, deletions or loss of function mutations in *ATG2B*, *ATG5*, *ATG12*, and *ATG9B* have been reported in different human tumors.^42^

Taken together, there are various studies indicating the involvement of impaired autophagy in tumor development. Also, autophagic cell death can play a potential tumor suppressor effect,^43^ which is discussed in a separate section.

Besides, several studies have suggested that the suppressor role of autophagy on the formation of neoplasia and cancer may be due to its inhibitory effects onnecrosis and inflammation.^19^, ^44^ In fact, cancer cells try to inactivate apoptosis to gain uncontrolled proliferation, and it seems that if autophagy in these cells is defective, they will undergo necrosis due to metabolic stress.^4^, ^45^ In other words, inactivation of autophagy induces necrosis in cells with defects in apoptosis pathways and exposed to metabolic stress, leading to the infiltration of inflammatory cells into tumor tissue. It has been reported that necrosis and inflammation are associated with increased tumorigenic potential of solid tumors.^46^ Therefore, autophagy contributes to cell survival in cancerous cells with defects in apoptotic pathways. Before angiogenesis occurs in a solid tumor, the inner cells are under different stressful conditions, where induction of autophagy causes the cells to pass through this condition. Accordingly, long‐term chronic necrosis is the fate of cancer cells with defective autophagy,^36^ indicating the momentous role of autophagy in tumor formation.

### 4.1 Autophagy and cell death

Although there are many studies on the protective role of autophagy in normal and cancer cells against stress conditions, the role of autophagy in cell death and tumor suppression has also been considered.^10^, ^34^, ^47^ Increased autophagosome formation in cells has been shown to induce cell death.^48^ For example, the accumulation of autophagic vacuoles in anti‐estrogen therapy with tamoxifen induces cell death in MCF‐7 cells. This autophagy‐induced cell death is a type of programmed cell death.^49^ Programmed cell death is divided into two categories: apoptotic cell death (as type І) and autophagic cell death (as type ІІ); there is also programmed necrosis (as type ІІІ).^50^ Autophagy‐induced cell death leads to tumor growth inhibition,^44^ probably due to the simultaneous occurrence of autophagy and apoptosis as long‐term stress and progressive autophagy can lead to cell death.^34^, ^44^ Also, induction of autophagy through up‐regulation of Beclin1 induces cell death.^51^ Down‐regulating Bcl2, an inhibitor of autophagy, has been reported to trigger cell death by increasing autophagy in HL60 leukemia cells.^52^ In addition, sorafenib, an autophagy inducer, induces autophagic cell death in renal cancer cell lines.^53^

Moreover, autophagy‐induced cell death is associated with other mechanisms of programmed cell death, which in turn can inhibit tumor progression. Activation of ULK1/2 transcription by p53 promotes autophagy and leads to cell death.^54^ So, ULK1/2 overexpression triggers cell death through increased autophagy.^55^ Decreased ULK1/2 expression has been reported in all grades of glioblastoma, therefore induction of autophagy appears to be helpful in suppressing brain tumors.^56^

Some studies have suggested that autophagy alone does not cause cell death but in combination with other death signals supports the progression of the cell death process. For example, DRAM1 exerts a modulatory role in autophagy mechanism and its signaling simultaneously with p53 signaling leads to cell death, indicating that autophagy is necessary but not sufficient for initiating and/or progressing cell death process.^57^ Various studies have also reported autophagic cell death as a result of apoptosis inhibition.^58^ Therefore, although increased autophagy has been associated with cell death, its relationship with induced cell death and apoptotic cell death needs further investigation.^7^

## 5 ROLE OF AUTOPHAGY IN TUMOR SURVIVAL AND PROGRESSION

Inconsistent with studies reporting the inhibitory roles of autophagy in tumorogenesis, there are several studies suggesting that autophagy may be associated with positive effects on tumor survival. Once the tumor has formed and increased in size, autophagy induces due to metabolic stress, hypoxia, and nutrient deficiency as well as high demand of inner tumor cells. Other tumor cells use autophagy to survive in the face of therapeutic interventions such as chemotherapy, radiation therapy, and hormone therapy. According to these studies, tumor cells rely on autophagy to survive especially by removing damaged organelles and proteins.^59^, ^60^ For example, eradicating damaged mitochondria prevents excessive ROS production and protects DNA from damage. Autophagy also maintains cellular metabolism by recycling the components required. Therefore, although cancer cells face a variety of stressful factors, autophagy may ensure cell survival by participating in the elimination of different stressful conditions.^29^ There are various reports that cancers such as pancreatic are “addicted” to autophagy in advanced stages to maintain energy homeostasis.^61^, ^62^ Also, autophagy increases in several types of cell lines and cancers even under nutrient‐rich conditions.^21^, ^59^

In addition, autophagy is involved in tumor cell metastasis. The motility of adhesive cancer cells is achieved by Epithelial‐to‐mesenchymal transition (EMT) and it has been reported that Transforming growth factor‐β (TGF‐β) and hypoxia‐related signaling pathways inducing EMT also induce autophagy.^63^

Moreover, defective autophagy can exert negative effects on the growth, progression, and metastasis of malignant cells.^24^ There are reports indicating inhibitory effects of defective autophagy on the invasion of lung and liver cancer cells.^64^, ^65^ Nevertheless, Catalano et al. demonstrated that induced autophagy down‐regulates transcription factors related to EMT in glioblastoma cells.^66^

Besides, cancer cell dormancy and chemotherapy resistance depend on the autophagic capacity of the cells.^4^ Cancer cells usually inactivate apoptosis pathways to increase survival and resistance to chemotherapy. Tumor cells with defective apoptosis enter dormancy under stress conditions and survive by inducing autophagy until they receive nutrients and return to normal conditions.^67^ This ability to survive, resist, and regenerate under stressful conditions can obstruct cancer treatment strategies.^34^

Oncogenic activity of oncogenes promoting tumor growth such as RAS and V‐Raf murine sarcoma viral oncogene homolog B (BRAF) seems to increase autophagy levels, indicating a link between autophagy and tumor progression.^68^, ^69^ Guo et al. reported that RAS‐driven cancer cells show increased levels of autophagy, and that this induction of autophagy activity appears to support the supply of metabolic needs under normal and starvation conditions of these cells.^68^ The role of autophagy in promoting tumor cell proliferation is more apparent with studies demonstrating inhibition of growth and progression in tumor cells with defects in autophagy.^21^ In this regard, inhibition of key proteins related to autophagy in human and mouse cell lines with RAS‐overexpression resulted in a decrease in the tumorigenic capacity of these cells,^68^, ^70^ suggesting a pivotal role for autophagy in the survival, growth and tumorigenicity of activated RAS‐driven tumors.

Autophagy has also attracted a lot of attention in developing therapeutic resistance. Inhibition of autophagy mediated by inhibitors or gene silencing causes cells to demonstrate limited ability to survive under stress conditions and restore homeostasis, leading to inhibition of tumor progression.^8^, ^21^, ^24^ Therefore, targeting autophagy by relevant inhibitors has been suggested in various studies, especially for the treatment of cancers that have been shown to be dependent on autophagy.^17^ Amaravadi et al. showed that inhibition of autophagy by drug or genetic manipulation led to a reduction in the resistance of isolated, cultured tumor cells to therapeutic agents compared to wild‐type cells.^71^ Also, Chen et al. reported increased autophagy during radiation therapy in tumor cells, which led to therapeutic resistance, and suggested that suppression of autophagy concomitant with radiation therapy may contribute to increased therapeutic efficacy.^72^ In addition, autophagy has been reported to play a role in preventing apoptosis in cancer cells. As mentioned above, cancer cells also employ autophagy to balance their metabolic rate and maintain their replicative power.^73^ For example, the increasing knowledge of the metabolome of prostate cancer (Pca) has led to a better elucidation of the pathways controlling the metabolism of PCa cells.^74^, ^75^ Autophagy has been found to be involved in the control of several cancer metabolic pathways as well as the regulation of apoptosis and prostate cancer progression.^73^ MAPK14 suppression has been reported to exert a significant effect on the growth of PCas, depending on the activity/inactivity of the STK11 protein. So that, drug‐mediated inactivation of MAPK14 does not affect the survival of STK11‐positive prostate cancer cells due to the activation of the AMPK‐dependent autophagic pathway. However, it induces apoptosis in STK11‐deficient prostate cancer cells. Also, blocking autophagy leads to induction of apoptosis in STK11‐positive cells treated with the MAPK14‐MAPK11 inhibitor. Thus, MAPK14 inhibition triggers a stress response in PCa cells that is buffered by AMPK‐dependent autophagy in STK11 positive cells but induces apoptosis in STK11‐deficient cells.^76^

The following is an overview of proteins and signaling pathways involved in autophagy:

### 5.1 Beclin1

Beclin1, encoded by the *BECN1* gene and an ortholog for the yeast *ATG6* gene, is a key protein involved in the beginning and nucleation steps of autophagy process.^8^, ^47^ Binding of Beclin1 to Vps34 through a conserved domain leads to the onset of autophagy. This domain also provides a tumor suppressionve activity for *BECN1*.^21^, ^27^ Although the *BECN1* gene is not considered a highly mutated gene in cancer (0.5% of all cancers), its monoallelic deletion can be seen in approximately 40% to 75% of ovarian, prostate, and breast cancers, which is why it is described as a “haploinsufficient tumor suppressor” gene.^44^, ^77^ Mouse models with *BECN1* allelic loss show an increased susceptibility to spontaneous lung adenocarcinoma, lymphoma, and liver carcinoma.^78^ Also, down‐regulation of *BECN1* has been reported in several cancers, including non‐small cell lung, brain, lymphomas, melanomas, and osteosarcomas.^79^, ^80^, ^81^, ^82^, ^83^ In mouse models, Beclin1 heterozygous dysfunction has been shown to be associated with increased susceptibility to malignancies by supporting the progression of precancerous to cancerous conditions.^40^ However, Li et al. demonstrated increased levels of Beclin1 in cancerous tissues of patients with colon cancer compared to surrounding normal tissues, and these increased expression levels were associated with favorable prognosis and increased survival.^84^

Beclin1 protein is regulated through interaction with B‐cell lymphoma 2 (Bcl2).^21^ Although, the early known role of Bcl2 family proteins in tumorogenesis is their modulatory roles in cell death, they are involved with key roles in various pathways of cell metabolism. As mentioned, it has been reported that one of the pathways for the negative effects of Bcl‐2 family proteins on autophagy is their interaction with Beclin1.^85^ Previous studies have shown that Beclin1 is inhibited under normal conditions through binding via the BH3 domain to Bcl2 or other anti‐apoptotic Bcl2 family proteins such as B‐cell lymphoma‐extra‐large (Bcl‐xL) and Bcl‐w. Under starvation conditions, Beclin1 is separated from its inhibitory proteins and can activate autophagy.^45^ Some proteins can induce autophagy by preventing the interaction between Bcl2 and Beclin1. For example, HMGB1 competitively binds to Beclin1 under starvation conditions and disrupts the interaction between Beclin1 and Bcl2.^86^ The Bcl2/Beclin1 complex is also regulated by stress‐activated signaling molecule c‐Jun protein kinase 1 (JNK1), so that stress‐activated JNK1 leads to the degradation of the Bcl2/Beclin1 complex by phosphorylating Bcl‐2.^87^

On the other hand, pro‐apoptotic members of the Bcl2 family such as BCL‐2 Associated agonist of cell death (Bad), Noxa, BCL‐2 interacting killer (Bik) and Bcl2/adenovirus E1B 19‐kDa interacting protein (BNIP3) contributes to autophagy induction.^88^ Therefore, usually increased expression of Bcl2 in cancer cells can inhibit autophagy process by binding to Beclin1.^89^ In addition, Bcl2 exerts suppressor effects on autophagy induced by elevated cytosolic calcium through activating its antagonist pathways.^90^

Interaction of some proteins with Beclin1, on the other hand, exerts positive effects on autophagy, such as Activating molecule in Beclin1‐regulated autophagy (AMBRA1), Bax interacting factor‐1 (BIF‐1), and UV radiation resistance‐associated (UVRAG), thereby inhibit cell proliferation and provides antitumor activity. Nevertheless, these proteins play other roles in addition to interfering with the autophagy pathway, which is beyond the scope of this study.^91^

### 5.2 UVRAG and Bif‐1

Tumor suppressor protein encoded by UV Radiation Resistance Associated Gene (UVRAG) plays a role in regulating the onset of autophagy as well as regulating the response to DNA damage.^92^ UVRAG exerts anti‐cancer effects through activating the Beclin1/PI3KIII complex and hence inducing autophagy.^93^ Destruction of the UVRAG chromosomal locus has been associated with a variety of malignancies, such as breast cancer.^94^ Also, monoallelic deletions or mutations in the *UVRAG* gene have been reported in colon cancers and gastric carcinomas.^95^, ^96^ The human colon cancer cell line HCT116 has a monoallelic mutation in the *UVRAG* gene that leads to significantly reduced expression levels of endogenous UVRAG and an anchorage‐independent cell growth. Additionally, heterozygous deletions in *UVRAG* gene increase the susceptibility of mouse models to cancer.^93^

Bif‐1 tumor suppressor, known as endophilin B1, induces autophagy by UVRAG‐mediated interaction with Beclin1.^31^, ^97^ Takahashi et al. showed an increased incidence of lymphoma and solid tumors in bif‐1^−/−^ mice compared to wild‐type models.^97^ Lee et al. reported decreased expression of Bif‐1 in gastric carcinoma cells compared to normal mucosal samples.^98^ Taken together these studies indicate that loss of function mutations in or depletion of autophagy regulators leads to tumorogenesis.

### 5.3 P62/SQSTM (sequestosome 1)

P62/SQSTM1 is a key receptor involved in selective autophagy.^99^ This protein binds to ubiquitinated cargoes such as protein aggregates through its ubiquitin‐binding domain.^45^ Also, P62/SQSTM1 can interact with LC3 located in the autophagosome membrane using its LC3‐interacting region (LIR) domain, leading to the cargo transfer into autophagosome. P62/SQSTM1 and transported cargo are destroyed in autophagosome through autophagy pathway.^100^

Defects in autophagy lead to the cell accumulation of p62,^100^, ^101^ mitochondrial damage, production of ROS, and thereby DNA damage. These consequences may affect predisposition to tumorogenesis.^102^ In addition, p62 knockdown aimed at eliminating its cell aggregates reduced ROS levels and DNA damage in cells with defective autophagy, and hence reduced defective autophagy‐induced toxicity and tumorogenesis.^102^, ^103^ Also, overexpression of p62 resulted in an increase in the ROS levels and tumor growth.^103^

Moreover, *P62* gene amplification was associated with renal cell carcinoma^104^ and P62^−/−^ mice have been shown to be resistant to Ras‐induced lung adenocarcinoma.^105^ Further, an association has been reported between defective autophagy and p62 accumulation in human tumors^106^ and there are several studies demonstrating p62 intracellular accumulation resulted from defective autophagy induces tumor progression in liver, lung and breast tissues.^107^, ^108^, ^109^ Taken together, while aggregates caused by p62 contributes to the cancer development, autophagy flux prevents tumorogenesis by limiting p62 intracellular accumulation.^19^

### 5.4 P53

P53 protein is encoded by *TP53* gene which is one of the most common mutated genes in human cancers (more than 50%).^35^, ^37^, ^85^ P53 plays key roles in regulating cell cycle checkpoints. Its activity can lead to cell cycle arrest, cellular senescence, and apoptosis, which introduces p53 as a tumor suppressor. In addition to involvement in cell cycle regulation, there are reports of p53 involvement in the regulation of autophagy.^35^, ^110^ Also, mutated *TP53* has been reported in many cancers and it has been suggested to induce tumorogenesis, possibly due to its key roles in the regulation of cell apoptosis and autophagy.^111^

**FIGURE 2:**
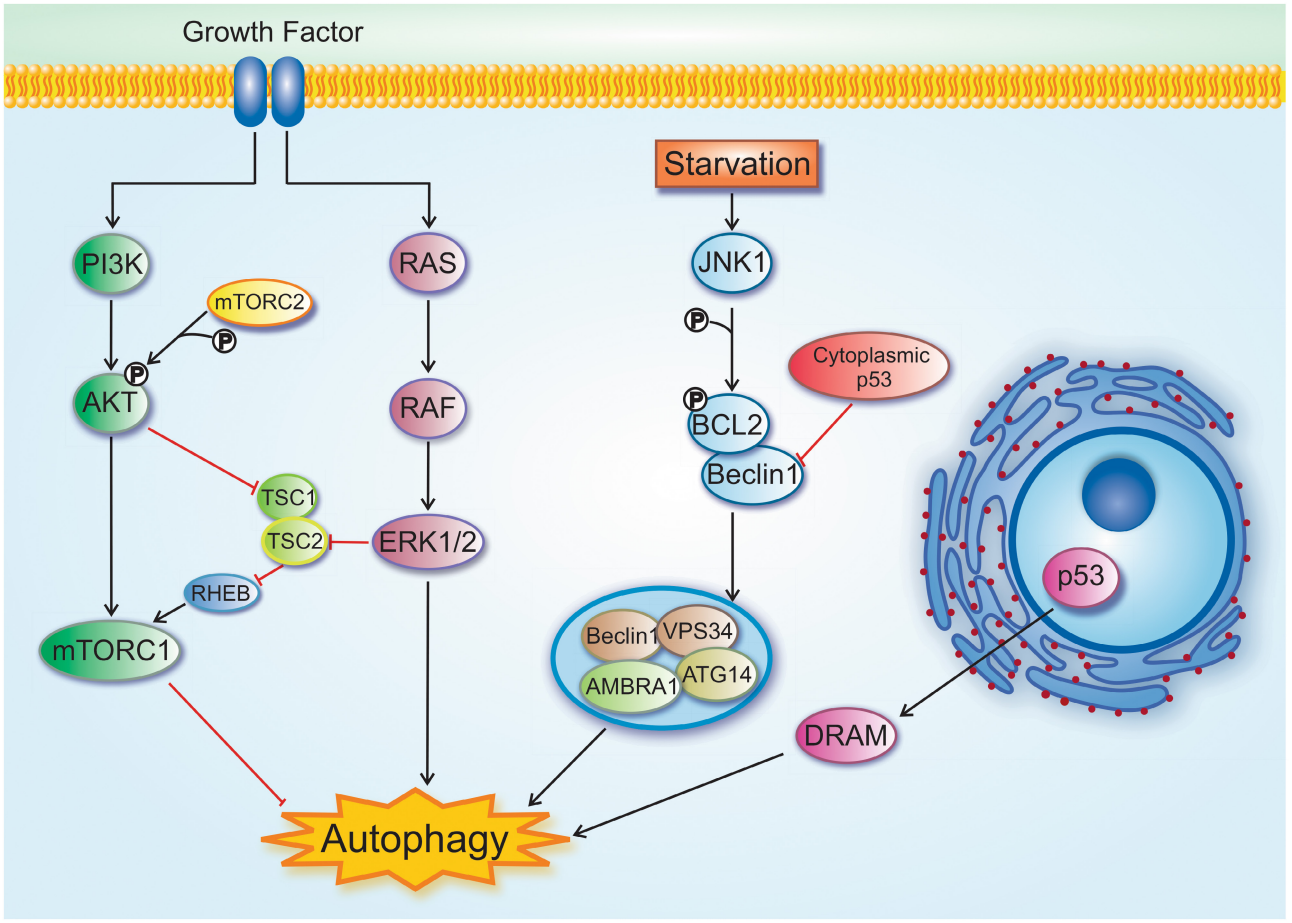
Overview of signaling pathways involved in the regulation of autophagy. (1) Growth factors and nutrients can induce PI3K, which activates AKT and subsequently mTORC1. Also, the activated AKT induce mTORC1 through TSC1/2 inhibition. (2) Growth factors inducethe Ras–Raf–MEK‐ERK1/2 signaling pathway. This pathway can directly induce autophagy. ERK complex can inhibit TSC1/2 complex, leading to activating mTORC1. (3) Under starvation and stress condition, Bcl2 is phosphorylated by JNK1 and thereby separated from Beclin1. Then, Beclin1 induces Vps34 complex formation, an important protein complex in the autophagy process. (4) P53 has different roles in autophagy. Under stress conditions, p53 nuclear localization promotes autophagy inducers such as DRAM and DAPK1. Nevertheless, cytoplasmic p53 leads to autophagy inhibition through Beclin1 and AMPK blocking.

P53 appears to play a paradoxical role in the autophagy pathway and its effects have been suggested to lead to cancer cell survival or death depending on its subcellular localization.^31^ Nuclear p53 induces transcription of autophagy‐related genes and thereby activates autophagy.^12^, ^112^ Under stress conditions, nuclear p53 induces autophagy through activating Damage‐regulated autophagy modulator (DRAM) or AMPK pathways, leading to inactivating mTOR pathway.^31^, ^55^, ^112^ Also, p53 can induce autophagy by activating Death‐associated protein kinase (DAPK), pro‐apoptotic Bcl2 proteins such as BCL‐2 Associated X (BAX), BAD, BNIP3 and p53 Upregulated modulator of apoptosis (PUMA), TSC2, and Sestrin1/2^31^, ^55^ (Figure 2). Gao et al. reported that p53 activates autophagy initiator proteins ULK1 and ULK2 in response to DNA damage to initiate autophagy and induce cell death.^54^ Inhibition of p53 has been shown to induce autophagy in enucleated cells and thereby its nuclear pool does not appear to be responsible for inhibiting autophagy. Instead, experimental studies have shown that cytoplasmic p53 can inhibit autophagy by blocking proteins involved in the autophagy pathway. Also, p53 depletion and autophagy initiation promote survival of p53‐deficient cells,^113^ possibly due to the maintenance of ATP levels under stressful conditions^31^ (Figure 2). There is an interconnection between pathways related to autophagy and p53 so that activated p53 induces autophagy and, on the other hand, induced autophagy results in a decrease in p53 activity levels.^114^ In addition, autophagy can promote tumor growth by inhibiting p53 in two ways: (a) through controlling stress and thereby inhibiting stress‐induced P53, as mentioned above. And (b) through providing raw materials for DNA replication and repair, and therefore, preventing DNA damage and damage‐induced p53 activation.^114^ Although one of the key mechanisms involved in tumor progression is P53 suppression by induced‐autophagy, it has been reported that autophagy also causes tumorogenesis through P53‐independent mechanisms that need to be further investigated.^114^

### 5.5 mTOR

The Mammalian target of rapamycin (mTOR) is the major regulator of autophagy.^23^ mTOR consists of two distinct multiprotein complexes with different functions called mTORC1 and mTORC2.^10^, ^23^, ^30^ In nutrient‐ and energy‐rich conditions, mTORC1 inhibits autophagy through binding to and phosphorylating ULK1 and thereby disrupts its interaction with AMPK. Under stress conditions such as hypoxia and starvation, ULK1 is separated from mTORC1 and induces autophagy.^115^ In addition, mTORC1 prevents autophagy by phosphorylating Atg13.^116^ The mTORC1 is inactivated by AMPK. Inhibition of mTORC1 and activation of AMPK induce autophagy.^8^, ^23^ Moreover, indirectly activation of mTORC1 by mTORC2 suppresses autophagy.^30^ Different single amino acid changes (gain of function mutations) in the components of mTOR have been reported in various human cancers.^117^

Rapamycin or rapalog drug is an autophagic inducer that promotes tumor survival in TSC mouse models through inhibiting mTOR pathway.^118^, ^119^ However, metformin has been reported to inhibit lymphoma and melanoma development by inducing autophagy and apoptosis mechanisms through AMPK activation and mTOR inhibition, and it has been associated with beneficial effects in pre‐clinical models.^120^, ^121^ Therefore, various studies showing inhibition of mTOR leading to induction of autophagy using various drugs emphasize the paradoxical effect of autophagy on tumor progression.^30^

### 5.6 HIF‐1α

Hypoxia is caused by defective angiogenesis, insufficient blood supply and high metabolic demand of tumor cells.^44^ Tumor cells exposed to Hypoxia induce hypoxia‐inducible factor (HIF) complex to overcome lack of oxygen.^122^ HIF‐1α, a subunit of HIF complex, is induced under hypoxia and regulates pathways involved in pH, metabolism, cell migration, inflammation, invasion, angiogenesis, and erythropoiesis, leading to adaptation of tumor cells to hypoxia conditions.^122^ Bellot et al. demonstrated that autophagy can be induced by hypoxia and HIF‐1α activity. HIF‐1α activates transcription of ATGs such as BNIP3 and BNIP3 like protein (BNIP3L, also known as NIX), resulting in initiating autophagy process through inhibiting interaction between Bcl2 and Beclin1.^123^ Under hypoxia, therefore, activation of HIF‐1α/BNIP3 pathway leads to induction of autophagy and thereby cell adaptation to hypoxic conditions.^122^ Hypoxia can also induce autophagy through HIF‐1α‐independent pathways, including inhibition of mTOR or AMPK activation.^124^, ^125^

### 5.7 BNIP3

Bcl2/adenovirus E1B 19‐kDa interacting protein 3 (BNIP3) is a pro‐apoptotic member of the Bcl2 family, with increased expression in hypoxic conditions.^126^ BNIP3 is involved in apoptosis and mitophagy induction.^127^ Increased expression levels of BNIP3 have been detected in the early stages of tumor progression. Nevertheless, it has been reported that BNIP3 is decreased in the advanced stages.^128^ Sowter et al. reported that BNIP3 expression levels in breast carcinoma cells were higher than that in normal adjacent cells.^129^

### 5.8 Signaling pathways

The following is an overview of some of the signaling pathways involved in autophagy:

1. The PI3K/Akt/mTOR signaling pathway, a negative regulator for autophagy, is regulated by AMPK, Phosphatase and tensin homolog (PTEN), Sirtuin1 (SIRT1), insulin, p38‐MAPK, p53 and ROS.^130^ Also, mTORC2 can inhibit the PI3K/AKT/mTOR pathway through AKT phosphorylation.^10^
2. Excessive activity of the Ras/Raf/ERK pathway; gain of function mutations in Ras or B‐Raf oncogenes, reported in various tumors, induces autophagy, which in turn supports tumor progression.^130^ Another study reported that ERK can active mTOR signaling by phosphorylating proteins like TSC2.^131^
3. Autophagy is also regulated by another signaling pathway called C‐Jun NH2‐terminal kinase (JNK) which induces autophagy by phosphorylating Bcl2 protein and inhibiting its interaction with Beclin1.^10^, ^37^, ^130^ JNK exerts a tumor suppressor function through constraining in vivo RAS‐induced cell transformation^37^ (Figure 2).
4. The intracellular signaling pathway mediated by calcium occurs in mitochondria, ER and lysosomes. Transporting Ca^2+^ is regulated by Inositol 1,4,5‐trisphosphate receptors (IP3Rs) or Two‐pore channels 1/2 (TPC1/2), Ryanodine receptors (RyRs), and Transient receptor potential superfamily pathways such as TRPML1 and TRPM2. Ca^2+^ transported into ER using IP3Rs can induce autophagy by activating Beclin1 or AMPK.^130^

In addition, signaling pathways regulating tumorogenesis are interconnected with autophagy signaling.^132^ For example, tumor suppressor genes such as PTEN, Liver kinase B1 (LKB1), TSC1, and TSC2 activate autophagy by inhibiting the mTOR signaling pathway.^132^, ^133^ Also, oncogenic proteins such as Bcl2, AKT, and Epidermal growth factor receptor (EGFR) can active mTOR signaling cascades and inhibit autophagy thus directly increase tumorogenesis.^133^

### 5.9 Autophagy and tumor angiogenesis

Although the relationship between autophagy and angiogenesis is still unclear and few studies have been performed in this field, the available reports indicate the involvement and importance of autophagy in angiogenesis.^36^

Angiogenesis is a biological process of creating new blood vessels from established capillary beds.^36^, ^134^ This process occurs in normal conditions such as organ development and wound healing as physiological angiogenesis and also in pathological conditions such as tumor growth and diabetic retinopathy.^36^ Angiogenesis provides oxygen and nutrient required for tumor cells, and is a protective toolfor tumor invasion, growth, and metastasis.^27^, ^134^ In fact, angiogenesis plays a key role in tumor growth and metastasis.^18^ The pathological angiogenesis occurs when the balance between pro‐ and anti‐angiogenic factors in tumor microenvironment shifts toward pro‐angiogenic factors.^134^ Angiogenic factors such as VEGF produced by tumor cells activate endothelial cells of surrounding capillaries and induce their sprouting and development of new blood capillaries into tumor.^27^, ^135^

One of the ways of autophagy involvement in the regulation of tumor angiogenesis has been reported to be interference in Vascular endothelial growth factor (VEGF) signaling.^35^ For example, under metabolic stress and hence increased autophagy, lysosomal degradation of neuropilin1 is induced, leading to inhibition of VEGF signaling and angiogenesis. Neuropilin1 is known as a pro‐angiogenic protein, and VEGF‐binding protein in endothelial and cancer cells.^136^ However, Liang et al. indicated that increased autophagy leads to accumulation of VEGF‐A through activating the JAK2/STAT3 pathway and thereby induces angiogenesis.^137^

Conflicting results have been reported for the relationship between increased autophagy and angiogenesis. Liang et al. demonstrated that rapamycin‐induced autophagy mediates angiogenesis in Human umbilical vein endothelial cells (HUVEC) through activating the AMPK/Akt/mTOR pathway.^138^ Conversely, inhibition of angiogenesis by increased autophagy has also been reported, and although it can indicate the paradoxical role of autophagy in the angiogenesis process, inducing autophagy in which may be a therapeutic target. For example, Sung et al. reported that Mebendazole induces autophagy in endothelial cells and in this way leads to inhibition of angiogenesis.^139^ Also, in vivo and in vitro investigations by Shinohara et al. demonstrated that mTOR inhibitors could inhibit angiogenesis, which in turn was associated with induced autophagy.^140^ In addition, chemerin, which is an angiogenesis inducer, resulted in induction of oxidative stress by increased ROS which in turn induced expression levels of ATGs in human aortic endothelial cells^141^ while angiogenesis inhibitors such as Endostatin and kringle5 induced autophagy levels in endothelial cells.^142^, ^143^

Anti‐angiogenic therapeutic strategies usually lead to the induction of starvation and hypoxia in cancer cells, which is associated with increased autophagy and thus increased angiogenesis. Hypoxia‐mediated autophagy causes tumor resistance to anti‐angiogenic drugs during cancer treatment,^27^, ^144^ and thereby, induced autophagy by cancer cells is an effective response for causing their resistance to anti‐angiogenesis therapies.^27^ Anti‐angiogenic strategies have been shown to induce autophagy flux by activating the HIF1α/AMPK signaling pathway, which promotes cell survival and drug resistance.^145^ Nevertheless, therapeutic strategies using a combination of anti‐angiogenic drugs and autophagy inhibitors seem to be more effective in cancer treatment,^146^ probably because apoptosis signaling pathways are also induced by these strategies.^147^

Besides, normalizing tumor vessels has been suggested as a cancer treatment strategy^36^ in order to restore perfusion, thereby reducing metastasis and increasing drug delivery.^134^ A relationship has been observed between tumor vascular normalization and autophagy, and it seems that different drugs with vascular normalization ability can affect autophagy. For example, chloroquine (CQ), a lysosomal inhibitor, can be effective in tumor vascular normalization.^148^

### 5.10 Targeting autophagy in cancer treatment

Autophagy in tumor cells under hypoxic, starvation, and other stress conditions is induced as an adaptive response and to maintain cell survival. Also, autophagy is activated in tumor cells as a response that induces cell resistance to treatment strategies such as chemotherapy and radiotherapy.^10^, ^21^ Therefore, it seems that the autophagic response of tumor cells acts as a defense mechanism to enhance adaptation to stress conditions and support the survival of remaining cells after exposure to therapeutic strategies.^149^

Therefore, applying autophagy inhibitors in combination with existing anticancer therapies seems to be an effective option for improving and/or developing treatment strategies, especially for cancers with high levels of and dependence on autophagy for survival under metabolic stress.^150^ In this regard, Amaravadi et al. reported that inhibition of autophagy in lymphoma mouse models increased the effectiveness of anti‐cancer drugs.^71^ Also, Levy et al. indicated that inhibition of autophagy leads to improved therapeutic outcomes in patients.^5^

In addition to various reports that increased autophagy leads to tumor cell resistance during anticancer therapy, there are other studies suggesting that many anticancer agents lead to cell death by increasing autophagy and that autophagy induction can act as a tumor suppressor strategy if cell apoptosis signaling is impaired.^149^, ^150^ Therefore, the latter suggest enhancing the mechanism of autophagy as an effective anticancer treatment option and highlight autophagy‐inducing anticancer drugs to increase tumor cell death.^8^ Nevertheless, it should be noted that inducing autophagy for cancer treatment may be a “double‐edged sword” because factors such as the type and stage of cancer and the type and duration of autophagy can affect its outcome.^34^ Overall, the choice of inhibiting or inducing autophagy as a cancer treatment strategy has been controversial and has faced many challenges due to its paradoxical functions. Table 1 presents a number of clinical trials targeting autophagy in various cancers.

**TABLE 1:** Autophagy modulators in clinical trial studies

Cancer Type	Drug	Effect on autophagy	Mode of action	Clinical trial phase	Identifer
Hepatocellular cancer	Rapamycin	↑	mTOR inhibitor	ІІ	NCT01079767
	Everolimus	↑	mTOR inhibitor	ІІІ	NCT01035229
	Sorafenib	↑	mTOR inhibitor	ІІІ	NCT00492752
Breast cancer	CQ	↓	Lysosome inhibitor	І, ІІ	NCT01023477
	HCQ	↓	Lysosome inhibitor	ІІ	NCT01292408
	CQ plus taxane	↓	Lysosome inhibitor	ІІ	NCT01446016
	HCQ plus ixabepilone	↓	Lysosome inhibitor	І, ІІ	NCT00765765
	PF‐04691502	↑	PI3K inhibitor	ІІ	NCT01430585
	Triciribine	↑		ІІ	NCT01697293
	Rapamycin plus trastuzumab	↑	mTORC1inhibitor	ІІ	NCT00411788
Colorectal cancer	HCQ plus FOLFOX and bevacizumab	↓	Lysosome inhibitor	І, ІІ	NCT01206530
	HCQ plus vorinostat	↓	Lysosome inhibitor	І, ІІ	NCT02316340
	Everolimus	↑	mTOR inhibitor	І, ІІ	NCT00522665
	MK‐2206	↑	AKT inhibitor	ІІ	NCT01186705
	Metformin plus 5‐FU	↑	AMPK activator	ІІ	NCT01941953
Ovarian cancer	Temsirolimus	↑		ІІ	NCT01460979
	Everolimus plus bevacizumab	↑	mTORC1 inhibitor	ІІ	NCT01031381
Pancreatic cancer	HCQ	↓	Lysosome inhibitor	ІІ	NCT01273805
	HCQ plus nucleoside analog gemcitabine	↓	Lysosome inhibitor	І, ІІ	NCT01128296
	NVPBEZ235	↑	PI3K inhibitor	ІІ	NCT01628913
	Metformin plus chemotherapy	↑	AMPK activator	ІІ	NCT01210911
Prostate cancer	HCQ	↓		ІІ	NCT00726596
	HCQ plus docetaxel	↓		ІІ	NCT00786682
	Vinblastine	↓		ІІ	NCT00003084
	Everolimus	↑	mTOR inhibitor	ІІ	NCT00657982
	Everolimus plus pasireotide	↑	mTOR inhibitor	ІІ	NCT01313559
	Everolimus plus paclitaxel	↑	mTOR inhibitor	І, ІІ	NCT00574769
	Metformin	↑	AMPK activator	ІІ	NCT01433913
Myeloma	HCQ plus rapamycin	↓		Early phase І	NCT01396200
Multiple myeloma	HCQ plus cyclophosphamide andbortezomib	↓		ІІ	NCT01438177
Multiple myeloma and plasma cell neoplasm	HCQ plus bortizomib	↓		І	NCT00568880
Acute myeloid leukemia	HCQ plus mitoxantrone and etoposide	↓		І	NCT02631252
Chronic myeloid leukemia	HCQ plus imatinibmesylate	↓		ІІ	NCT01227135
Multiple myeloma	HCQ plus rapamycin, cyclophosphamide and dexamethasone	↓		І	NCT01689987

Abbreviations: 5‐FU, 5‐Fluorouracil; CQ, chloroquine; HCQ, hydroxychloroquine.

## 6 LIVER CANCER

Liver tissue cells are dependent on autophagy due to its specific physiology, and paradoxical functions of autophagy (inhibitory or supportive) have been reported HCC.^151^, ^152^

### 6.1 Tumor‐inhibiting autophagy in HCC

There are various studies suggesting an inhibitory role for autophagy in the development and progression of chronic liver disease and liver cirrhosis, with defective autophagy being a primary cause of HCC.^153^ In this regard, loss of function mutations in ATGs and hence inhibition of autophagy led to the onset or progression of hepatocellular carcinoma, indicating importance of regulating basal autophagy in liver tissue.^154^ Takamura et al. reported that mice with deletions in *ATG5* and *ATG7* and thereby defective autophagy developed multiple liver tumors.^38^ Also, the incidence of spontaneously occurring tumors such as HCC was observed in *BECN1* heterozygous knockout mice, while homozygous knockout mice died during embryogenesis.^78^ In addition, p62 accumulation were observed in steatohepatitis and HCC mouse models with *BECN1* loss of function mutations.^155^ In a study by Bao et al., increased p62 accumulation was observed in liver tumor cells compared with surrounding normal cells.^156^ Various studies have investigated p62 accumulation effects on tumor formation and progression in several cancers such as HCC and pre‐malignant liver disease.^55^ Umemura et al. reported that overexpression of p62 in liver cells may provide a poor prognosis for HCC and induce liver tumorogenesis.^157^ Also, specific knockdown of P62 expression level was associated with reduced tumor size in mice with Atg7 deficiency in hepatocytes, which is consistent with contribution of p62 accumulation in hepatic tumor formation.^38^ NF‐κB‐related signaling pathways seem to be involved in liver tumorogenesis resulted from p62 accumulation.^155^ In addition, knockdown of the PI3K/AKT/mTOR pathway has been reported to prevent HCC cell proliferation and migration through inducing autophagy, suggesting the tumor suppressor roles of autophagy in liver.^158^ In support of this suggestion, it is observed that arenobufagin, *a natural bufadienolide derived from toad venom*, prevents HCC cell growth through suppressing the PI3K/AKT/mTOR pathway and thereby inducing autophagy and apoptosis in the cells.^159^

### 6.2 Tumor‐inducing autophagy in HCC

As mentioned, autophagy contributes to tumor cell survival by maintaining cell homeostasis. There are various reports that different tumors such as HCC maintain their survival through autophagy.^151^ Autophagy is increased in advanced liver cancer which is associated with increased tumor malignancy and low survival rates in HCC patients. Also, there is a reported direct relationship between the rate of tumor progression and the levels of autophagy, so that the rate of autophagy is higher in the advanced stages of HCC.^160^, ^161^ Wu et al. demonstrated LC3B overexpression in HCC samples compared to surrounding normal samples. The expression levels of LC3B were associated with vascular invasion, lymph node metastasis, and poor prognosis.^162^ In addition, overexpression of ULK1 was observed in HCC cancer cells by Xu et al.^163^ Another study on liver carcinoma indicated that autophagy induces HCC cell invasion by activating EMT.^65^ EMT in HCC cells may be induced by autophagy through cAMP/PKA/CREB signaling and induction of TGF‐β.^164^

### 6.3 Induction of autophagy in HCC as a therapeutic option

The PI3K/Akt/mTOR pathway is a cell cycle regulatory signaling pathway that controls several vital functions such as growth, survival, and metabolism.^151^ There are several reports that mTOR activity is increased and autophagy is inhibited in HCC patients (15%–41%) which are associated with tumor cell survival, suggesting targeted mTOR pathway inhibition as a therapeutic option for HCC.^151^, ^165^ Rapamycin (sirolimus) is an mTOR kinase suppressor that is used as an autophagy inducer with anti‐proliferative and anti‐tumor effects.^166^ A phase ІІ clinical trial study on 25 patients with advanced HCC showed rapamycin exerts anti‐tumor effects by inhibiting mTOR and inducing autophagy.^167^ Moreover, everolimus (RAD001) as a rapamycin derivative demonstrated anti‐tumor activities in HCC pre‐clinical studies.^168^ In addition, inhibition of mTOR by everolimus and a PI3K/mTOR signaling inhibitor which have synergistic effects on each other resulted in a reduction in tumor size in HCC mouse models through an increase in autophagy/mitophagy levels^169^ (Table 2). Also, induction of autophagy has been used to increase cell death in the treatment of HCC.^151^ In this regard, sorafenib has been suggested as a treatment option for HCC patients, with the observation that sorafenib resulted in induced autophagy‐dependent cell death in vitro and in vivo HCC models.^170^ Also, combination therapy with sorafenib and an autophagic inhibitor drug improved efficiency of advanced HCC treatment through autophagy‐mediated cell death. Sorafenib also induced autophagy‐mediated cell death in treatment‐resistant HCC patients.^171^ Yang et al. demonstrated that sorafenib inhibits liver cancer cell proliferation through mediating autophagy and cell apoptosis. They also reported suppressed cell apoptosis in combined use of CQ with sorafenib.^172^ Additionally, Hidvegi et al. showed reduced hepatic fibrosis in alpha‐1 antitrypsin deficient mice using pro‐autophagic agents.^173^

**TABLE 2:** A summary of the autophagy modulators in cancer treatment

Cancer type	Drug	Effect on autophagy	Drug development status	Ref.
Hepatocellular cancer	Everolimus	↑	In vivo	^168^
	Sorafenib	↑	In vivo and in vitro	170, 171, 172
	CQ plus sorafenib	↓	In vivo and in vitro	174, 177
	CQ plus cisplatin CQ plus 5‐FU	↓	In vivo and in vitro	^175^
	CQ plus oxaliplatin	↓	In vivo and in vitro	^176^
	3‐MA plus sorafenib and vorinostat	↓	In vitro	^178^
	CQ plus bevacizumab	↓	In vivo and in vitro	^179^
Breast cancer	Everolimous	↑	In vitro	^195^
	HCQ plus tamoxifen	↓	In vivo and in vitro	^200^
	Resveratrol plus rapamycin	↓	In vitro	^201^
	3‐MA	↓	In vitro	^206^
Colorectal cancer	Torin‐1	↑	In vitro	^225^
	Aspirin	↑	In vitro	^226^
	AZD‐2014	↑	In vivo and in vitro	^227^
	Bufalin	↑	In vitro	^228^
	Quercetin	↑	In vitro	^231^
	Resveratrol	↑	In vitro	^232^
	3‐MA plus 5‐FU	↓	In vivo ad in vitro	^233^
	CQ plus 5‐FU	↓	In vitro	^234^
	CQ plus oxaliplatin CQ plus bevacizumab	↓	In vivo and in vitro	^237^
	3‐MA plus oxaliplatin Baf A1 plus oxaliplatin	↓	In vitro	^238^
Ovarian cancer	Quinacrine	↑	In vivo and in vitro	^256^
	Resveratrol	↑	In vitro	^257^
	MORAB‐003	↑	In vivo and in vitro	^258^
	Arsenic trioxide plus everolimus	↑	In vivo and in vitro	^260^
	Elaiophylin plus cisplatin	↓	In vivo and in vitro	^263^
	Ellagic acid plus doxorubicin	↓	In vitro	^265^
Hematological cancers	Arsenic trioxide	↑	In vitro	271, 272, 273
	Fludarabine	↑	In vitro	^275^
	Dexamethasone	↑	In vitro	276, 277
	Idarubicin	↑	In vitro	^278^
	Everolimus	↑	In vivo	^279^
	Resveratrol	↑	In vitro	^281^
	CQ plus cytarabine BafA1 plus cytarabine	↓	In vitro	^283^
	CQ plus SAHA	↓	In vitro	^285^
	CQ plus interfrone‐1	↓	In vitro	^286^

Abbreviations: 5‐FU, 5‐Fluorouracil; 3‐MA, 3‐Methyladenine; Baf A1, Bafilomycin A1; CQ, chloroquine; HCQ, hydroxychloroquin.

### 6.4 Inhibition of Autophagy in HCC as a therapeutic option

Based on the protective role of autophagy in stress conditions such as those are caused under cancer treatment, many researchers have focused on autophagy inhibition for treating some tumors such as HCC.^151^

One of the drugs with favorable results used for treating advanced HCC is sorafenib, which inhibits tyrosine kinase receptors.^153^ Sorafenib can also initiate cell autophagy through suppressing the mTOR pathway, which in turn leads to the cell accumulation of autophagosomes and cell resistance to treatment.^174^ Due to the autophagy‐induced cell resistance to chemotherapy, several studies have suggested that autophagy inhibition may provide HCC cell sensitivity to chemotherapy.^154^ Accordingly, autophagy inhibitors such as CQ and Hydroxychloroquine (HCQ) have been developed. These drugs suppress autophagy by preventing lysosome‐to‐autophagosome fusion and are also used for treating malaria.^151^ Combined use of autophagy inhibitors with tumor suppressor drugs such as sorafenib, cisplatin and 5‐Fluorouracil (5‐FU) has been reported to improve therapeutic effects.^174^, ^175^ Moreover, treatment of xenograft models and HCC cell lines, respectively, with oxaliplatin in combination with CQ and sorafenib in combination with CQ led to increased effectiveness of the tumor suppressants^176^, ^177^ (Table 2). Also, Yuan et al. reported increased therapeutic effects of sorafenib for HCC treatment using its combination therapy with 3‐Methyladenine (3‐MA), an autophagy inhibitor, or a small interfering RNA (siRNA)‐specific for inhibiting Beclin1.^178^ Further, Guo et al. indicated that bevacizumab prevents growth of xenograft tumors and triggers increased autophagy. They reported enhanced effects of bevacizumab in combination with CQ as well as increased tumor cell apoptosis.^179^

Based on the type of autophagy function (supportive or suppressive) in HCC progression, autophagy modulation provides new prospects for the treatment of liver cancer.^153^ Although there is evidence suggesting the high potential of autophagy modulation as a therapeutic strategy for HCC, the clinical application of these autophagy modulators needs further investigation.^151^

## 7 BREAST CANCER

Paradoxical function of autophagy has also been reported in breast cancer, on the basis of which new perspectives have been proposed for the treatment of this cancer.

### 7.1 Tumor‐inhibiting autophagy in breast cancer

Emerging evidence suggests autophagy as a factor with suppressive roles in the early stages of breast tumors. Several studies have reported tumorogenesis induced by defective autophagy in breast tissue.^180^ Also, monoallelic deletions of Beclin1 which is a key Atg have been reported in different breast tumors.^41^, ^181^ Li et al. indicated decreased mRNA and protein levels of Beclin1 caused by monoallelic deletion or epigenetically silencing of the gene in breast cancer samples.^182^ In addition to the findings that Beclin1 is a tumor suppressor in breast and ovarian cancers probably due to its deletion in 40%–75% sporadic breast and ovarian cancers, there are studies suggesting that deletion of *BECN1* gene may be due to its proximity to *BRCA1* gene and not to its tumor inhibitory functions and *BRCA1* deletions cover *BECN1* chromosomal position.^24^, ^91^ In fact, gene deletions *BECN1* in breast and ovarian cancers have been shown to be associated with gene deletions *BRCA1*, suggesting that *BECN1* deletions may not be the main cause of breast tumor development and hence the proposed tumor inhibitory function for Beclin1 is controversial^183^ However, Liang et al. demonstrated decreased proliferation and tumorogenesis of MCF‐7 cells after Beclin1 overexpression.^41^ It has been also reported that Beclin1 deficiency in breast cells could contribute to tumor formation in a way independent of its role in autophagy and through interaction with Bcl2. This observation was highly regarded because Bcl2 exhibits increased expression levels in breast and many other cancers (more than 50%).^184^ Moreover, down‐regulation associated with cancer progression of other ATGs such as ULK1 was also observed in breast tumor samples.^185^

### 7.2 Tumor‐inducing autophagy in breast cancer

Autophagy in many solid tumors such as breast cancers contributes to tumor cell survival and invasion.^186^ There have also been reports of autophagy involvement in breast cancer cell dormancy. The leading cause of death in breast cancer is recurrence of the disease due to metastasis, which is due to the activation of dormant, migrated breast tumor cells in other tissues. These cells supply their energy needs during dormancy period through autophagy.^187^ In a study including 489 patients with breast carcinoma, Beclin1 exhibited higher expression levels in triple‐negative breast cancer samples than other types.^188^ Also, Gong et al. reported higher levels of autophagy as well as higher expression levels of Beclin1 in breast cancer stem‐like cells compared to nontumor cells. They also indicated that autophagy is an essential process for survival and tumorogenicity of breast cancer stem cells.^189^ In addition, Zhao et al. demonstrated increased LC3B expression level in triple‐negative breast cancer patients which was correlated with increased tumor metastasis and decreased patient survival rate.^190^ Furthermore, the deletion of *FIP200*, another ATG, reduced tumor formation and progression in the MMTV‐PyMT mouse model of breast cancer.^191^

### 7.3 Induction of autophagy in breast cancer cells as a therapeutic option

Although several studies suggest that inhibition of autophagy may be helpful in the treatment of breast cancer, there are a number of studies suggesting that autophagy may play a role in cell death, and hence inducing autophagy during breast cancer treatment seems to be helpful.^150^ For example, Bcl2 inhibition in MCF7 cells resulted in autophagy induction and thereby autophagy‐mediated cell death. These observations were disappeared through suppressing *ATG5*, suggesting the involvement of induced autophagy in the cell death mechanisms. In addition, autophagy‐related toxicity effects increased through using some drugs simultaneously with Bcl2 inhibition combination. For example, autophagy is induced using low doses of doxorubicin, while its high doses lead to cell apoptosis. It has been reported that the combined use of Bcl2 siRNA and low doses of doxorubicin enhanced autophagy‐mediated cell death and tumor inhibition in MCF‐7 cell line.^192^ Wilson et al. reported that Vitamin D sensitizes breast cancer cells to radiation therapy through autophagy induction.^193^ Different studies have shown that using mTOR inhibitors leads to sensitivity to radiation and autophagy‐induced cell death in breast cancer cell lines.^194^, ^195^

### 7.4 Inhibition of Autophagy in breast cancer as a therapeutic option

Autophagy can be induced during anti‐cancer therapy and acts as an enhancing factor of tumor cell survival and resistance to treatment. From this perspective, targeted suppression of autophagy has been highlighted in the treatment of breast cancer.^196^ In several studies, simultaneous use of autophagy inhibitors and tumor‐inhibiting drugs improved anti‐tumor therapeutic effects. For instance, it has been reported that autophagy is induced during anti‐estrogen therapy for ER^+^ breast cancer, and therapeutic effect of tamoxifen is augmented if autophagy is also inhibited.^197^ Also, inhibition of autophagy in the breast cancer cell lines exhibited remarkable effects on the treatment of tamoxifen‐resistant cells.^198^, ^199^ Moreover, the anti‐tumor effects of tamoxifen on the inhibition of ER^+^ breast cancer cells were enhanced in a combination therapy of tamoxifen with HCQ^200^ (Table 2). The favorable effects of concomitant use of autophagy inhibitors with other tumor inhibiting drugs have also been investigated. For example, a combination therapy of rapamycin with resveratrol suppressed autophagy and induced cell apoptosis in ER‐positive and negative breast cancers.^201^ Several reports have highlighted autophagy as a mechanism involved in the development of trastuzumab resistance in the treatment of HER2‐positive breast cancer.^202^ HER2‐positive breast cancer cells with Beclin1 deficiency have been demonstrated to be susceptible to trastuzumab therapy.^203^ Additionally, knockdown of *ATG12* led to overcoming cell resistance to trastuzumab therapy and suppressing tumor growth in trastuzumab‐resistant xenografts.^204^

Treatment of MCF7 cells with epirubicin induced autophagy which in turn protected cells from apoptosis. However, autophagy inhibition enhanced epirubicin toxicity effects through increased cell apoptosis.^205^ Further, 3‐MA reduces breast cancer cell survival through autophagy inhibition.^206^ Treatment of breast cancer cell line MDA‐MB‐231 with autophagy inhibitors such as 3‐MA or CQ resulted in increased cell sensitivity to radiation and reduced survival of cells exposed to radiation.^207^ Nevertheless, 3‐MA increased autophagy flux in nutrient‐rich conditions.^208^

Although more evidence suggests that inhibiting autophagy combined with anti‐cancer therapeutic strategies is a promising way to treat breast cancer, cell death mediated by induced autophagy is a challenge that needs to be more considered in some cancer therapies.^150^

## 8 COLORECTAL CANCER

The paradoxical functions of autophagy in colorectal cancer (CRC) have also been considered,^209^ which are discussed here.

### 8.1 Tumor‐inhibiting autophagy in CRC

Defective autophagy‐related genes have been shown to be closely associated with colorectal tumorogenesis.^210^, ^211^ For example, *ATG5* gene deletions or mutations of autophagy‐related tumor suppressor gene *URAG* have been frequently observed in colorectal cancer.^93^, ^212^ Approximately, 23% of CRC patients exhibit *ATG5* gene deletions, which play a key role in the growth of intestinal cancer.^212^ A comparison between colorectal cancer mice models Apc^Min^ with mice models with heterozygous deletions of *Atg5* (*Atg*5^+/−^) and also Apc^Min^ mice with *Atg5* ^+/+^ showed an increase in the number and size of adenomas in *Atg*5^+/−^ mice.^213^ In addition, monoallelic mutations of the *UVRAG* gene, which is an autophagy regulator relevant to BECN1 pathway, is frequently detected in human CRC.^93^ Mutated *UVRAG* leads to decreased autophagy and increased CRC cell proliferation and metastasis.^214^ Activation of the PI3K–AKT–mTOR pathway and hence inhibition of autophagy has also been reported in CRC.^211^

### 8.2 Tumor‐inducing autophagy in CRC

The importance of autophagy functions in the development and progression of CRC has been suggested in various in vivo studies.^209^ Wu et al. reported increased expression levels of Beclin1, LC3, and mTOR in colorectal cancer cells, which the increased LC3 expression levels exhibited a positive correlation with increased Beclin1 and cell differentiation and negative correlation with mTOR expression levels. They showed that increased autophagy may be associated with increased tumorogenesis ability of CRC cells. Moreover, increased expression levels of mTOR contributed to proliferation and lymph node metastasis.^215^ Further, it has been reported that LC3‐ІІ exhibits increased expression levels in colorectal cancer samples compared to normal colorectal tissues in particular in advanced stages.^216^

There are observation suggesting paradoxical roles for *BECN1* in CRC; supportive and suppressive functions on tumorogenesis ability and growth of CRC cells.^210^ Beclin1 expression level has been reported to increase in advanced tumor stages of CRC.^217^ Li et al. showed that Beclin1 expression levels are increased in colorectal cancer cells compared with normal adjacent cells.^84^ Also, increased Beclin1 expression showed a positive correlation with distant metastasis. These observations highlight Beclin1 as a protein with tumor supportive functions in colorectal cancer.^218^ Moreover, rectal adenocarcinoma patients with low expression levels of *BECN1* respond better to treatment than patients with higher levels of *BECN1* expression.^219^ Nevertheless, although increased *BECN1* expression has been observed in colorectal cancer patients (95%) in patients with gastric cancer (83%),^220^ other solid tumors such as ovarian, lung, and liver cancers have not shown an increase in *BECN1* expression levels. In contrast to the first studies, Beclin1 is expressed in normal tissues and there are in cancer cells various reports of reduced and no expression of Beclin1 in solid tumors other than colorectal and gastric.^221^, ^222^, ^223^ For example, decreased expression levels of *BECN1* are observed in glioblastoma multiform and high‐grade brain tumors.^224^

Moreover, a study by Lauzier et al. showed differential in vivo responses of colorectal cancer cells to autophagy inhibition‐mediated by CQ. They also demonstrated that although CRC cells need autophagy to proliferate, in vitro autophagy inhibition was associated with differential responses in different colorectal cancer cell lines. Caco‐2/15 and HTC116 cells exhibited sensitivity to autophagy inhibition, while proliferation and tumorogenicity of SW480 and LoVo cells increased in response to autophagy inhibition.^209^

### 8.3 Induction of autophagy in CRC as a therapeutic option

Several studies have suggested autophagy induction as an effective therapeutic strategy for CRC. In CRC patients with decreased *BECN1* expression, activation of autophagy by enhancing *BECN1* expression may be effective in treatment.^216^ Also, inducing autophagy by inhibition of mTOR has been effective in different studies on CRC. For example, in vitro treatment of colorectal cancer stem cells with Torin‐1, an mTOR inhibitor, decreased cell survival, and tumor inhibiting effects of Torin‐1 suggested its efficacy in CRC treatment.^225^ Moreover, other mTOR inhibitors such as aspirin, a non‐steroidal anti‐inflammatory drug, and AZD‐2014 exhibited anti‐tumor effects through autophagy‐mediated cell death in CRC.^226^, ^227^ It is also noted that autophagy‐induced cell death is effective in particular in apoptosis‐resistant CRC cells. Bufalin, an apoptosis inducer in several human tumor cell lines, induced autophagy‐mediated cell death in colon cell lines through activating the MAPK/JNK pathway which participates in Atg5 and Beclin1 up‐regulation as well as increasing ROS production^228^ (Table 2). Furthermore, B‐group soyasaponins caused autophagy‐induced cell death in human HCT‐15 colon cancer cells.^229^

Besides, Zhan et al. reported that MS‐275, a synthetic benzamide derivative of Histone deacetylase inhibitor (HDAC), led to Atg7 overexpression in human colon cancer cells, and subsequently induced autophagy and apoptosis.^230^ Similarly, treatment of Ha‐Ras‐transformed human colon cells with quercetin induced autophagy and exhibited anti‐tumor effects.^231^ Induction of apoptosis through increased ROS accumulation has also been reported in the use of resveratrol, an anti‐cancer agent, and its effects exhibited a correlation with increased LC3‐ІІ levels in human colon cancer cells.^232^

### 8.4 Inhibition of autophagy in CRC as a therapeutic option

Several studies suggest inhibition of autophagy as an effective therapeutic strategy in colorectal cancer. In this regard, it has been reported that inhibition of autophagy can promote effects of 5‐FU, a chemotherapy medication. Treatment of CRC cell lines with 5‐FU resulted in an increase in LC3‐ΙΙ and autophagy, and subsequently a reduction in cell death. However, cell apoptosis increased by adding 3‐MA or CQ, which inhibits autophagy by preventing autophagosome formation. In fact, 3‐MA and CQ potentiated the anti‐tumor effect of 5‐FU on colon cancer cells.^233^, ^234^ Also, simultaneous use of 5‐FU and radiotherapy enhanced autophagy and subsequently treatment resistant in CRC cell lines. Nevertheless, cell treatment with CQ in combination with radiation therapy or chemotherapy resulted in increased cell apoptosis.^235^ In addition, photodynamic therapy is used as a treatment for patients with colorectal cancer. Xiong et al. suggested that CQ in combination with photosan‐ІІ‐mediated photodynamic therapy can enhance apoptosis in CRC cell lines.^236^ Furthermore, another study revealed that CQ in combination with anti‐angiogenic drugs such as oxaliplatin and bevacizumab sensitizes colon cancer cells.^237^

Oxaliplatin is used as a treatment for metastatic colorectal cancer but cells become resistant through increasing autophagy. Shi et al. reported increased oxaliplatin‐induced cell death through autophagy inhibitors such as 3‐MA, Bafilomycine A1, and RNAi‐mediated knockdown of essential ATGs such as Atg5 and Beclin1.^238^ It has been reported that, although inhibition of autophagy in *Apc* ^*min/+*^mice inhibits colorectal tumor growth through a CD8‐mediated immune response, it induced intestinal dysbiosis.^239^ Moreover, *BECN1* and *UVRAG* have been demonstrated to protect CRC cells against radiation‐induced DNA breaks and cell death by maintaining genomic integrity.^240^

Recent studies have found that autophagy modulator drugs exhibit an improved efficiency for CRC treatment, in use alone or in combination with other therapeutic agents. Nevertheless, further research is needed.^210^, ^211^

## 9 OVARIAN CANCER

In addition to the above‐mentioned cancers, autophagy plays paradoxical roles in ovarian cancer, which may be dependent on cancer cell conditions such as nutrient deficiency, hypoxia, and chemotherapy.^241^

### 9.1 Tumor‐inhibiting autophagy in ovarian cancer

Various studies have suggested that down‐regulation of autophagy increases ovarian cancer cell survival.^242^ As mentioned previously, *BECN1* gene deletion is observed in 40%–75% of ovarian cancers.^41^ A study by Valente et al. demonstrated that type І ovarian carcinoma exhibits high levels of Beclin1 compared to type ІІ. Also, Beclin1 was detected with low or no expression levels in 18 of 20 patients with grade ІІІ ovarian cancer.^243^ In addition, Beclin1 showed low expression levels in malignant epithelial cells of ovarian cancer than ovarian benign, borderline tissue. Also, Beclin1 expression levels exhibited an inverse correlation with higher stages of ovarian tumors. Moreover, LC3 exhibited increased expression levels in ovarian benign, borderline hyperplastic tissue compared to malignant ovarian cancer. Nevertheless, LC3 expression levels in stages ІІІ and ІV FIGO were lower than stages І and ІІ.^244^ Patients with higher expression of Beclin1 and LC3 exhibited a better response to treatment.^245^ Another study reported that patients with higher levels of Beclin1 showed better survival than patients with low levels of Beclin1 expression.^246^ Lin et al. indicated that decreased expression levels of Beclin1 can be a biomarker for ovarian tumor cell malignancy. Moreover, ovarian carcinoma patients with high expression levels of Bcl‐xL, an autophagy inhibitor, and low expression levels of Beclin1 represented low survival rates. The decreased expression level of Beclin1 was also associated with poor prognostic of ovarian carcinoma.^247^ Furthermore, high expression of cytoplasmic p62 has been observed in ovarian cancer cells with an association with development of serous carcinoma and tumor, indicating expression levels of cytoplasmic p62 as a biomarker for ovarian cancer.^248^ It has also been reported decreased expression levels of other ATGs in ovarian cancer cells such as *DRAM2*.^249^

### 9.2 Tumor‐inducing autophagy in ovarian cancer

A study on ovarian cancer has shown that Beclin1 expression is increased in malignant ovarian tumors compared to its benign tumors.^250^ Another study reported increased levels of Beclin1 expression levels in endometrioid adenocarcinoma, and also suggested that high expression levels of Beclin1 may be due to hypoxia. increased Beclin1 expression level was correlated with tumor grade as well.^251^

Spowart et al. demonstrated that clear cell ovarian cancer cell lines exhibit high expression levels of LC3A under hypoxic conditions. Also, LC3A showed higher expression in stage ΙΙΙ compared to stage Ι or ΙΙ. Nevertheless, these findings were not observed in other ovarian cancer cells; indicating that autophagy exerts different roles in different subtypes of ovarian cancer.^252^ It has also been reported that there is a correlation between induced autophagy and ovarian cancer cell dormancy are linked. Lu et al. indicated that re‐expression of *ARHI* gene, which known as a tumor suppressor and with down‐regulated expression in ovarian cancer, induces autophagy and subsequently dormancy in ovarian cancer cells xenografted in mice.^253^

### 9.3 Induction of autophagy in ovarian cancer as a therapeutic option

Various studies have suggested that autophagy and cell apoptosis are closely related to each other. Considering the relationship between signaling pathways involved in autophagy and function of autophagy in cell apoptosis, it has been shown that autophagy is activated by inhibition of Ras/MAP and PI3K/AKT/mTOR pathways, leading to induced ovarian cancer cell cycle arrest.^254^ Also, Zhou et al. demonstrated that Tanshinone І enhances autophagy and cell apoptosis in ovarian cancer cells through inhibiting the PI3K/AKT/mTOR pathway.^255^ According to a study by Khurana et al., quinacrine cell treatment promoted autophagy in ovarian cancer cell line OvCa and induced autophagy‐mediated cell death.^256^ Also, Lang et al reported induced autophagy and cell apoptosis in human ovarian tumor cell lines OVCAR‐3 and caov‐3 using resveratrol, which triggers ATG5 expression and promotes LC3 cleavage. They also observed reduced OVCAR‐3 cell mortality‐induced by resveratrol when they simultaneously treated OVCAR‐3 cells with resveratrol and CQ, an autophagy inhibitor.^257^ In addition, expression induction of autophagy‐related genes *BECN1* and *LC3‐ІІ* has been reported using farletuzumab (MORAB‐003), which is a humanized anti‐folate receptor alpha monoclonal antibody.^258^ LC3‐ІІ expression has been demonstrated to be induced using dihydroptychantol 2 as well. Dihydroptychantol 2 also inhibits the growth and development of ovarian cancer cells SKOV3.^259^ Further, Wen et al. showed that treatment of different human ovarian cancer cell lines with MORAB‐003 led to autophagy induction. Also, the MORAB‐003‐induced autophagy induced caspase‐3‐mediated cell death and inhibition of ovarian tumor cell growth.^258^ Liu et al. indicated that simultaneous cell treatment with arsenic trioxide, an autophagy inducer, and everolimus led to induced autophagy and apoptosis in xenografted ovarian cancer cells and also exerted a synergistic effect on tumor inhibition.^260^

### 9.4 Inhibition of autophagy in ovarian cancer as a therapeutic option

As mentioned, ovarian cancer cells can acquire resistance to chemotherapy through increasing autophagy. Also, various studies have shown that inhibition of autophagy can be effective in the treatment of ovarian cancer.^241^ For example, treatment of human ovarian cancer with cisplatin induces autophagy, which in turn causes cell resistance. These findings suggest increased efficacy of anti‐tumor chemotherapy agents in combination with autophagy inhibitors. Further, inhibition of autophagy using Beclin1 siRNA led to increased cisplatin‐induced ovarian cancer cell apoptosis.^261^, ^262^ Another study indicated that simultaneous cell treatment with cisplatin and elaiophylin, an autophagy inhibitor, decreased ovarian cancer cell viability^263^ (Table 2). Tang et al. reported that the sensitivity of ovarian cancer cell lines to cisplatin is augmented through LC3B inhibition.^264^ In addition, ellagic acid potentiates the anti‐cancer effects of doxorubicin in ovarian cancer cells through inhibiting autophagy.^265^ Pagotto et al. showed that autophagy plays a protective role in ovarian cancer stem cells. In fact, higher levels of autophagy are observed in ovarian cancer stem cells compared to their non‐stem counterparts. In their study, the viability and in vivo tumorogenic capacity of these cells were reduced using CQ and Crisper/cas9 system designed for inhibition of *ATG5*.^266^

Nevertheless, although many studies demonstrated that inhibition of autophagy in ovarian cancer cells exerts protective effects in favor of increased cell survival, there are several reports that autophagy induction promotes ovarian cancer cell malignancy.^242^ Therefore, the findings of various studies suggest that autophagy manipulation may be useful in the treatment of ovarian cancer and off course that the protective or suppressive function of autophagy should be considered. Also, this proposed therapeutic strategy needs to be investigated in various clinical trials.^241^

## 10 HEMATOLOGICAL CANCERS

Several studies have reported the critical function of autophagy in blood cancers. Autophagy plays a role in controlling differentiation and self‐renewal of hematopoietic stem cells (HSCs). Human HSCs cultured show high levels of autophagy, which appears to be involved in supporting differentiation and self‐renewal.^267^ For example, inhibition of autophagy mediated by 3‐MA or its attenuation via Atg5 shRNA resulted in the failure of HSCs to differentiate into neutrophils and colony formation in vitro, indicating a loss of HSC stemness properties due to autophagy deficiency.^268^ Also, *ATG7* has been suggested as a crucial regulator inadult HSC maintenance. Deletion of *ATG7* has been shown to affect HSC function and cause severe DNA damage in mice as well as abnormal myeloproliferation. In addition, lymphoid and myeloid production is significantly impaired in ATG7‐deficient mice.^269^ Liu et al. reported that the HSC numbers were significantly reduced in liver of mouse embryo as a result of conditional deletion of FIP200 through Tie2‐Cre. The capacity of mouse embryonic hepatocytes to undergo multi‐lineage regeneration was clearly reduced after FIP200 deletion following injection of the inducer polyinosine‐polycytosine, explaining the disappearance of the embryonic compartment for HSCs.^270^ An increase in the incidence rate of hematologic malignancies such as lymphoma has also been reported with autophagy defects in HSCs. There are reports that Beclin1 haploinsufficiency is involved in the development of lymphoma and lymphoproliferative disease.^40^

These findings suggest a critical role for autophagy in supporting the maintenance and differentiation of HSCs, as defective autophagy can lead to myeloproliferative disorders and cancer development.

### 10.1 Induction of autophagy in hematological malignancies as a therapeutic option

There are reports of cytotoxic effects of various anticancer drugs through autophagic cell death for use in hematologic malignancies. For example, arsenic trioxide (As_2_O_3_) showed strong anticancer effects by inducing autophagic cell death in leukemic cell lines and primary leukemia progenitors of myeloid leukemia patients.^271^, ^272^, ^273^ Fludarabine is a nucleoside analog commonly used in treatment of chronic lymphocytic leukemia (CLL). It prevents DNA synthesis and DNA repair. According to Mahoney et al., fludarabine was introduced for activating autophagy in CLL cells. However, the fludarabine‐induced cell death was not affected by autophagy suppression in these cells.^274^ Fludarabine has also been reported to promote BECN1‐dependent autophagy through downregulation of MCL‐1 expression leading to autophagic cell death in fludarabine‐sensitive leukemic B cells.^275^ Dexamethasone has been shown to induce cell death in lymphoid leukemia and multiple myeloma. Several studies have reported that dexamethasone‐induced autophagic cell death is mediated by promyelocytic leukemia protein overexpression and promyelocytic leukemia protein‐dependent Akt dephosphorylation.^276^, ^277^ Idarubicin‐induced autophagy is known as a pro‐death mechanism in leukemic cells. Idarubicin inhibits mTOR by inducing the activity of its upstream inhibitor, AMPK, or by decreasing the activity of its activator, Akt, which causes autophagic cell death. However, there are reports that the cytotoxicity of idarubicin in REH cells, a human leukemic cell line, is partially reduced by pharmacological autophagy impairment, Bafilomycin A1 or CQ.^278^ The mTOR inhibitor everolimus caused cell death by triggering autophagy in acute lymphoblastic leukemia cells in an in vivo model.^279^ Induction of autophagy in multiple myeloma cell lines was also observed with FTY720 treatment, which could be an explanation for increased cell death. So, simultaneous treatment of these cells with FTY720 and bafilomycin A1, an autophagy inhibitor, led to increased cell viability compared to treatment of cells with FTY720, suggesting that the induction of autophagy by FTY720 is involved in multiple myeloma cell death.^280^ Moreover, Resveratrol induced autophagic cell death in chronic myelogenous leukemia cells by up‐regulating p62 through JNK and activating AMPK.^281^

### 10.2 Inhibition of autophagy in hematological malignancies as a therapeutic option

Cancer cells use autophagy inorder to adapt to the challenging tumor microenvironment and stress or damage caused by treatment. For example, chemotherapy is often associated with the induction of autophagy, which leads to reduced therapeutic effects and increased drug resistance. Therefore, restoration of tumor cell sensitivity is a promising insight for hematological malignancies.^282^ In these cases, there are suggestions that combination therapies using autophagy inhibitors with targeted chemotherapy drugs are effective for hematological malignancies. The anti‐leukemia drug cytarabine has been shown to enhance AMPK/ERK phosphorylation, inhibit Akt, and reduce mTOR activity, thereby leading to cytoprotective autophagy in leukemic cells. Due to increased cell death resulting from accumulation of DNA breaks, mitochondrial damage, and oxidative stress resulting from drug‐induced (bafilomycin A1 and CQ) or genetic‐induced (depletion of LC3 or p62 by RNA interference) autophagy defects, combined use of cytarabine and autophagy inhibitors was developed as a therapeutic strategy for leukemia.^283^

About 50% of hematological malignancies show dysregulated tumor suppressor p53. This protein is involved in controlling cytoprotective autophagy.^284^ In the absence of p53, metabolic stress induced by treatment with the histone deacetylase inhibitor suberoylanilide hydroxamic acid (SAHA) induces autophagy, which promotes chronic myeloid leukemia cell survival. Furthermore, SAHA enhances anticancer efficacy when autophagy is inhibited by the autophagy inhibitor CQ.^285^ Zhu et al. demonstrated that autophagy also exerts pro‐survival effects in chronic myeloid leukemia treated with interferon. The JNK1‐STAT1 and NF‐ҝB pathways were both activated by interferone‐1, which increased BECN1‐ATG‐ATG5, a key regulator of autophagy pathway. Also, the ability of Chloroquine to inhibit autophagy improved the cytotoxic effects of interfrone‐1.^286^ Some clinical studies on the combined use of CQ and HCQ with conventional therapies as therapeutic strategies for hematologic malignancies are listed in Table 1.

Altogether, the effects of autophagy manipulation as a therapeutic option for hematological malignancies can be different depending on the type of treatment applied, supporting cell survival or cell death. Nevertheless, further studies are needed to clarify the mechanisms controlling cell density.^284^

## 11 CONCLUSION

According to the above‐mentioned findings in different subsections, autophagy manipulation as a cancer therapeutic strategy should be considered from two perspectives. In several advanced cancers, cell survival is dependent on autophagy, and therefore cancer cells exposed to treatments such as chemotherapy and radiation induce autophagy, which in turn leads to suppression of anti‐cancer effects of therapeutic agents and also causes cell resistance. However, increased autophagy following the use of anti‐cancer agents results in the autophagy‐mediated cell death in several cancers. Therefore, inducing or inhibiting autophagy depends on the origin of the cancer cells, the type of cancer, and the behavior of the cancer cells exposed to treatment. For this reason, it is necessary to determine the basal levels of autophagy in various cancers. Nevertheless, measuring the basal levels of autophagy in human tumor samples is difficult, and current knowledge about in vivo evaluating autophagy is incomplete. At present, activity levels of autophagy are assessed through detecting LC3 by using Western blotting or immunohistochemistry and tracking accumulated autophagic vesicles. Overall, increasing our knowledge of the mechanisms involved in autophagy and discovering more accurate strategies for measuring autophagy levels could open up new perspectives on cancer treatment, although it is a major challenge for clinical cancer treatment.

## AUTHOR CONTRIBUTIONS

**Farnaz Ahmadi‐Dehlaghi:** Data curation (equal); investigation (equal); validation (lead); writing – original draft (lead). **Parisa Mohammadi:** Conceptualization (equal); investigation (equal); supervision (equal); writing – original draft (equal). **Elahe Valipour:** Validation (equal); writing – review and editing (lead). **Pouya Pournaghi:** Writing – review and editing (equal). **Sarah Kiani:** Visualization (equal); writing – review and editing (equal). **kamran mansouri:** Conceptualization (lead); supervision (lead); validation (equal).

## CONFLICT OF INTEREST STATEMENT

The authors declare no conflict of interest.

## Data Availability

Data sharing is not applicable to this article as no new data were created or analyzed in this study.
